# Assessing the Effects of Climate on Host-Parasite Interactions: A Comparative Study of European Birds and Their Parasites

**DOI:** 10.1371/journal.pone.0082886

**Published:** 2013-12-31

**Authors:** Anders Pape Møller, Santiago Merino, Juan José Soler, Anton Antonov, Elisa P. Badás, Miguel A. Calero-Torralbo, Florentino de Lope, Tapio Eeva, Jordi Figuerola, Einar Flensted-Jensen, Laszlo Z. Garamszegi, Sonia González-Braojos, Helga Gwinner, Sveinn Are Hanssen, Dieter Heylen, Petteri Ilmonen, Kurt Klarborg, Erkki Korpimäki, Javier Martínez, Josue Martínez-de la Puente, Alfonso Marzal, Erik Matthysen, Piotr Matyjasiak, Mercedes Molina-Morales, Juan Moreno, Timothy A. Mousseau, Jan Tøttrup Nielsen, Péter László Pap, Juan Rivero-de Aguilar, Peter Shurulinkov, Tore Slagsvold, Tibor Szép, Eszter Szöllősi, Janos Török, Radovan Vaclav, Francisco Valera, Nadia Ziane

**Affiliations:** 1 Laboratoire Ecologie, Systematique et Evolution, Unité Mixte de Recherche 8079 Centre National de la Recherche Scientifique-Université Paris-Sud XI-AgroParisTech, Batiment 362, Université Paris-Sud XI, F-91405 Orsay, France; 2 Departamento de Ecología Evolutiva, Museo Nacional de Ciencias Naturales, Consejo Superior de Investigaciones Cientificos, C/José Gutiérrez Abascal 2, Madrid, Spain; 3 Departamento de Ecología Funcional y Evolutiva, Estación Experimental de Zonas Áridas, Estacion Experimental de Zonas Aridas-Consejo Superior de Investigaciones Cientificos, Ctra. Sacramento s/n, La Cañada de San Urbano, Almería, Spain; 4 National Museum of Natural History - Sofia, Bulgarian Academy of Science, Sofia, Bulgaria; 5 Departamento de de Anatomía, Biología Celular y Zoología, Universidad de Extremadura, Badajoz, Spain; 6 Section of Ecology, Department of Biology, FIN-20014 University of Turku, Finland; 7 Departamento de Ecología de Humedales, Estación Biológica Doñana (Estacion Biologica de Doñana-Consejo Superior de Investigaciones Cientificos), Sevilla, Spain; 8 Cypresvej 1, DK-9700 Brønderslev, Denmark; 9 Departamento de Ecología Evolutiva, Estación Biológica Doñana (Estacion Biologica de Doñana-Consejo Superior de Investigaciones Cientificos), Sevilla, Spain; 10 Max Planck Institute for Ornithology, Eberhard-Gwinner-Straße, Haus Nr. 11, Seewiesen, Germany; 11 Norwegian Institute for Nature Research – Norwegian Institute for Nature Research, Polar Environmental Centre, Tromsø, Norway; 12 Evolutionary Ecology Group, Department of Biology, University of Antwerp, Antwerpen, Belgium; 13 Division of Genetics and Physiology, University of Turku, Finland; 14 Skovvej, DK-9490 Pandrup, Denmark; 15 Departamento de Parasitología, Universidad de Alcalá, Alcalá de Henares, Spain; 16 Faculty of Biology and Environmental Sciences, Cardinal Stefan Wyszynski University in Warsaw, Woycickiego 1/3, Warsaw, Poland; 17 Departamento de Zoología, Facultad de Ciencias, Universidad de Granada, Granada, Spain; 18 Department of Biological Sciences, University of South Carolina, Columbia, South Carolina, United States of America; 19 Espedal 4, Tolne, DK-9870 Sindal, Denmark; 20 Evolutionary Ecology Group, Hungarian Department of Biology and Ecology, Babeş-Bolyai University, Cluj Napoca, Romania; 21 Department of Biology, Centre for Ecological and Evolutionary Synthesis, University of Oslo, Oslo, Norway; 22 Institute of Environmental Sciences, College of Nyíregyháza, H-4400 Nyíregyháza, Sóstói út 31/b, Hungary; 23 Behavioural Ecology Group, Department of Systematic Zoology and Ecology, Eötvös Loránd University, Pázmány Péter stny 1/C, Budapest, Hungary; 24 Institute of Zoology, Slovak Academy of Sciences, Dúbravská cesta 9, Bratislava, Slovakia; 25 Department of Biology, Faculty of Science, Badji Mokhtar University, Boite Postal 12, Annaba, Algeria; Centers for Disease Control and Prevention, United States of America

## Abstract

**Background:**

Climate change potentially has important effects on distribution, abundance, transmission and virulence of parasites in wild populations of animals.

**Methodology/Principal Finding:**

Here we analyzed paired information on 89 parasite populations for 24 species of bird hosts some years ago and again in 2010 with an average interval of 10 years. The parasite taxa included protozoa, feather parasites, diptera, ticks, mites and fleas. We investigated whether change in abundance and prevalence of parasites was related to change in body condition, reproduction and population size of hosts. We conducted analyses based on the entire dataset, but also on a restricted dataset with intervals between study years being 5–15 years. Parasite abundance increased over time when restricting the analyses to datasets with an interval of 5–15 years, with no significant effect of changes in temperature at the time of breeding among study sites. Changes in host body condition and clutch size were related to change in temperature between first and second study year. In addition, changes in clutch size, brood size and body condition of hosts were correlated with change in abundance of parasites. Finally, changes in population size of hosts were not significantly related to changes in abundance of parasites or their prevalence.

**Conclusions/Significance:**

Climate change is associated with a general increase in parasite abundance. Variation in laying date depended on locality and was associated with latitude while body condition of hosts was associated with a change in temperature. Because clutch size, brood size and body condition were associated with change in parasitism, these results suggest that parasites, perhaps mediated through the indirect effects of temperature, may affect fecundity and condition of their hosts. The conclusions were particularly in accordance with predictions when the restricted dataset with intervals of 5–15 years was used, suggesting that short intervals may bias findings.

## Introduction

The commonest interactions between species occur between parasites and their hosts [1], [2]. These interactions depend on parasite prevalence and abundance, rate and mode of transmission, effect of parasites on fecundity and mortality of hosts and the level of anti-parasite defence by hosts [3]. Each of these different steps in the interaction can potentially be affected by environmental conditions including climatic conditions [4]. Several studies have indicated that parasite diversity is higher at low latitudes [5]–[7], and negative impacts on avian [8] and human hosts [5], [9], [10] are higher at low latitudes. This is partly linked to latitudinal clines in climate, but also clines in host immunity and host population density [11]–[13], and consequently climate may have direct effects on interactions between hosts and parasites by affecting the abundance of parasites, or by indirectly affecting phenology of parasites and hosts [14].

Many studies have pondered the effects of climate change on parasites, host-parasite interactions and ultimately veterinary and public health [15]–[22]. Increasing temperature is the main factor linked to climate change effects on living organisms although changing precipitation and wind may also play a role. Many parasites are advancing their date of emergence [4], [23]–[25], and some that were previously active only in the summer are now active year-round [26], [27]. A longer and earlier period of reproduction by parasites may increase the number of parasite generations per year, as a longer breeding season of the host provides parasites with a selective advantage [28], [29]. However, a longer reproductive season may also allow hosts to better defend themselves against parasites and hence achieve higher reproductive success because timing of reproduction is less constrained by migration [30], [31]. Most models of the effect of climate change on host-parasite interactions predict that the distribution of parasites will move northwards, while the overall geographical range of the parasite will not change, nor will total population size [4]. Host populations may also expand as a consequence of climatic change, although that will partly depend on their immunity and hence their ability to cope with novel parasite assemblages [32].

Empirical assessments of effects of climate change on host-parasite interactions are scarce, and, therefore, there is high uncertainty in predicting the consequences. Møller [33] analyzed prevalence and intensity of several species of parasites exploiting the barn swallow *Hirundo rustica*, showing that parasites that do not complete their life cycles on hosts were more strongly affected by climate change than those that do. In addition, a directly transmitted mite seemed to have reduced its virulence in response to climate change, while cell-mediated immunity of swallows decreased over time. A recent meta-analysis concluded that the incidence of blood parasites and avian malaria increased during recent decades [34]. Likewise, changes in prevalence of a brood parasitic cuckoo were related to change in temperature [35]–[36]. In addition, several studies have shown changes in phenology of both hosts and parasites [34]. Finally, some studies of hosts and parasites have demonstrated changes in parasite-mediated mortality and resistance of hosts [34], [36], [37]. For example, host races of the cuckoo *Cuculus canorus* rely on migratory hosts that now arrive well before the arrival of the parasitic cuckoo. Therefore such host races have declined dramatically in abundance in recent years [36]. While there is evidence of changes in host-parasite interactions related to climate change, there is considerable unexplained heterogeneity among parasite taxa.

In an attempt to fill this gap, we established a European network of scientists interested in host-parasite interactions exploiting existing historical data on abundance and impact of parasites on their bird hosts. By returning to exactly the same host populations in 2010, and recording parasitological, host demographic and host density data as already done in exactly the same way in an earlier study year, we developed a paired design to test for climate-driven change in host-parasite interactions. We explicitly tested for an effect of interval between first and second study year on temperature and parasite and host variables while statistically controlling for the time elapsed between studies. Such within-population comparisons are known to be particularly powerful because they allow separation of within from between population variance [38]. Despite the statistical advantage of this approach and the common use of among-year variation for exploring effects of climate changes [39]–[42], we are unaware of any previous study adopting this design to explore the effects of time for such a broad range of species and populations.

The objectives of the study were to test the link between climate change and host-parasite interactions. Specifically, we tested (1) if prevalence and mean abundance of parasites and abundance of hosts have changed over time within study sites; (2) if the changes in parasite prevalence and abundance are related to changes in temperature within study sites; (3) if the change in prevalence and abundance of parasites has fitness consequences for their hosts; and (4) if a decrease in population density of hosts can be predicted by an increase in abundance of parasites and an increase in temperature. We obtained data on 17,891 individuals of 89 parasite populations for 24 different species of hosts making this study by far the largest ever conducted on the relationship between climate change and host-parasite interactions.

## Methods

### Ethics Statement

This study was carried out in strict accordance with current laws of all the countries where the study was performed and following the recommendations of the ‘Guidelines to the Use of Wild Birds in Research’ (J. Fair, E. Paul and J. Jones, eds. 2010. Ornithological Council, Washington, D. C.). All efforts were made to ameliorate suffering of animals and minimize handling time. No birds were injured or killed during the study.

Blood sampling was carried out at locations in Belgium, Bulgaria, Denmark, Finland, Hungary, Norway, and Spain. The sampling consisted of collection of a tiny blood sample from the brachial vein. This procedure is a standard procedure performed by numerous bird banders throughout the world for the study of avian malaria. No birds showed signs of negative consequences of blood sampling. We have recorded as high survival rates from sampled birds as from other birds. Collection of blood was specifically authorized in each location under the permits issued for this work.

### Study Populations

**Figure 1:**
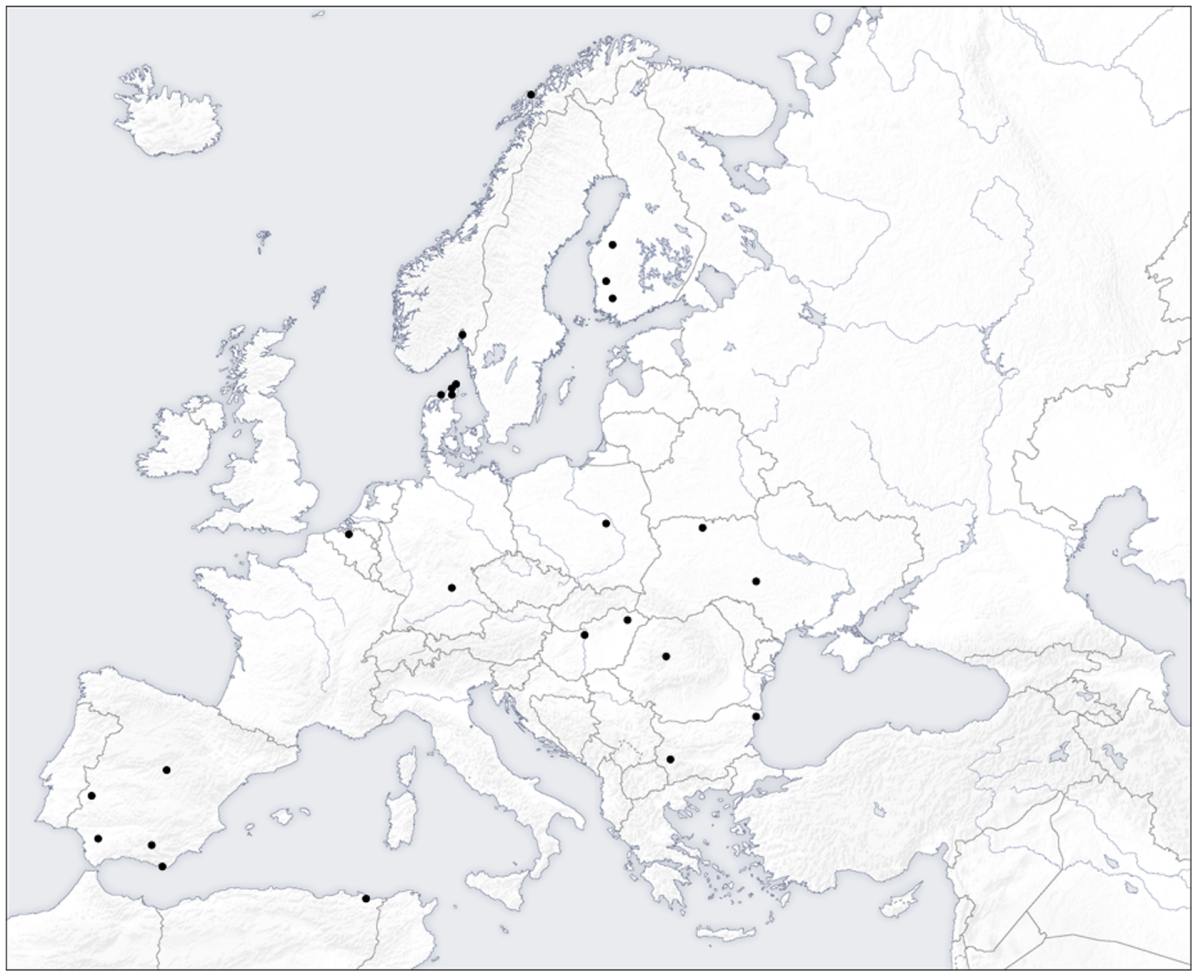
Geographical distribution of the 26 sites for the study of temporal change in abundance and prevalence of parasites of birds and their consequences.

In January 2010 we requested 58 European scientists, who had published on bird-parasite interactions to participate in the project. Participants were mainly selected based on a recent review of the effects of bird parasites on the fitness of their hosts [8]. Three requests were made and eventually 37 scientists participated in the study. Some scientists did not participate because they were not working on parasites any more, or they were committed to other projects during 2010. The geographical distribution of the study sites is shown in Fig. 1. We deliberately collected all recent samples during a single breeding season (2010) to reduce potential bias related to variation in variables among years. Although this approach may reduce variation in estimated differences due to the second year being the same for all populations, geographic variation in climatic conditions during 2010 was sufficiently large among study populations to ensure conclusions that are independent of the particular climatic conditions of 2010. Geographic variation in temperature in 2010 did not differ from that of the first study year (Levene's test, *F* = 0.45, d.f. = 1, 88, *P* = 0.50), although the first study year differed among populations. We asked all participants to use **exactly** the same methods in 2010 as during the first year of their study, but also that the same person conduct or at least supervise the study, thus ensuring that all studies were consistent in methodology over time to avoid inter-observer variability.

### Parasites

We distinguished between four functional parasite groups based on their taxonomic and transmission status: protozoans including blood parasites, feather parasites, dipteran parasites and non-dipteran parasites. Protozoans are generally vector-transmitted. Feather parasites such as chewing lice and feather mites live in the plumage of birds. Dipteran parasites include louseflies and blowflies. Non-dipteran parasites included blood-sucking fleas, ticks and mites. When more than one taxon of parasite within one of these groups were investigated in a target host species and population, we estimated mean values, which were used for subsequent analyses. Variance explained by the four functional groups of parasites was significantly larger than the residual variance (that was explained by different kinds of parasites within the four functional groups) for parasite abundance (*F* = 4.31, d.f. = 3,44, *P* = 0.01) and prevalence (*F* = 4.14, d.f. = 3,82, *P* = 0.009), which further supports the use of mean values.

We requested information on prevalence (the fraction of adults or nests harboring a given parasite) and the mean abundance of parasites exactly as done in the first year of the study and again in 2010. We requested that participants record information on demographic traits and population density of hosts as described below.

Information on the methods used to quantify parasites can be found in the references in Møller et al. [8] and Merino et al. [43]. In brief, we quantified prevalence and intensity of infection (mean abundance of parasites per host/host nest). Most studies either used two independent methods to quantify parasites or quantified parasites repeatedly for the same individuals/nests to allow estimation of repeatability. All studies used statistically significant and highly repeatable estimators of parasite prevalence and abundance.

### Temperature Trends

We used the E-OBS gridded dataset (version 5.0) maintained by the European Climate Assessment and Dataset (ECAandD) (http://eca.knmi.nl/) to estimate temperatures at the study sites [44]. We calculated mean monthly temperature for each 0.25×0.25 degree squares, covering the study sites from the daily mean temperature of the E-OBS gridded dataset. Change in temperature during the month best covering the laying, incubation and nestling period of each bird host species was estimated as the temperature in 2010 minus the temperature in the first study year divided by the interval between the two years. This change in temperature over time (°C/year) is referred to as change in temperature throughout the remainder of this paper.

### Life History and Population Density of Hosts

We requested that all participants record for each host the date of laying of the first clutch, clutch size, reproductive success (no. fledglings) and body condition (estimated as body mass at the start of the breeding season, if possible). We also requested a local estimate of population density of hosts (such as the proportion of nest boxes occupied for nest box studies, the total number of occupied nest boxes, the number of individual birds captured, colony size, or in a few cases population density for open nesting species). The entire dataset is reported in File S1 in Tables S1–S2, while Table S3 in file S1 presents correlations between prevalence and response variables in the supplementary material (ESM).

### Statistical Analyses

All variables were log_10_-transformed to achieve approximately normal distributions. The effects of time between sampling of host populations and parasite intensity and prevalence were explored by Repeated Measures ANOVAs with laying date, clutch size, brood size, body condition of adults, population density, parasite loads and parasite prevalence estimated for the two study years as within subject, and population identity, host (or parasite) identity, latitude, and temperature change per year (°C/years) as between subject factors. All analyses were performed individually in order to avoid inclusion of more than one interaction term of between and within factors (i.e. repeated measure).

The effects of parasitism on characteristics of host populations were also explored using Repeated Measures ANOVAs with laying date, clutch size, brood size, body condition of adults and population density estimated for the two study years as within subject factors and change in parasite abundance and prevalence as between subject factors. Parasite functional group, latitude and temperature change (°C/years) were also included in the models as additional between factors to statistically control for the effect of these factors on changes in host population characteristics. A significant interaction between repeated measure and between factors would be consistent with an effect of the latter on change in host population characteristics.

Statistical analyses assume that each observation provides equally precise information about the deterministic part of the process variance [38]. Sample sizes differed among species due to differences in abundance of hosts, and such variation may affect the precision of estimates and hence the outcome of the statistical analyses [45], [46]. Therefore, we weighted all analyses by sample size to ensure that individual observations contributed relative to their precision.

The findings might depend on duration of the interval between first and second study year. Therefore, we conducted a second series of analyses based on datasets with an interval of 5–15 years, and these results are reported in Tables S4–6 in File S1. All analyses were performed with Statistica 10 [47]. Values reported are means (SE).

## Results

### Summary Statistics

The mean change in temperature per year across the 89 data sets was + 0.085°C (SE = 0.029), with a range from −0.65°C to +1.07°C, differing significantly from zero which would be expected if temperature on average was the same in the first and the last year of study (t-test, *t* = 2.90, *P* = 0.0047). A similar increase in temperature over time was found for studies based on intervals of 5–15 years (+0.076°C (SE = 0.020), with a range from −0.55°C to +0.50°C). Temperature in the first year was lower than in the second year (*t* = 2.52, *P* = 0.013), and that was even more so for studies with an interval of 5–15 years (*t* = 4.30, *P*<0.0001). The number of years between the first study year and 2010 was on average 10 years (SE = 0.63), range 1 to 39 years with 78% within the interval 5–15 years. We also detected a large range in change in temperature across studies from −2.94°C to +4.04°C, on average +0.40°C, for the mean period of study. Because change in temperature between the first year of study and 2010 decreased slightly with the number of years of study (*F* = 4.02, d.f. = 1,87, *r* ^2^ = 0.04, *P* = 0.048, estimate (SE) = −0.053 (0.027)), implying that temperatures decreased more in studies of long duration, we used temperature change among study years divided by number of years elapsed between the first year and 2010 in the subsequent analyses. There were no additional effects of latitude or longitude on change in temperature (latitude: *F* = 0.05, d.f. = 1,85, *P* = 0.83; longitude: *F* = 0.39, d.f. = 1,85, *P* = 0.54), suggesting that these variables were not confounding effects on change in temperature.

Total sample size was 9935 hosts (or host nests depending on study) in the first year and 7956 in 2010, or in total 17,891 hosts/nests. A total of 37.2% of the 89 parasites studied were ectoparasites, and 76.4% of the 89 parasites lived permanently on the host, with the remaining species living apart from the host at ambient temperature at least part of the annual cycle. Prevalence and abundance of parasites in the first study year was independent of the duration of the study period (GLM: prevalence: log-likelihood χ^2^ = 2.12, d.f. = 1, *P* = 0.14; abundance: log-likelihood χ^2^ = 0.42, d.f. = 1, *P* = 0.51).

### Temporal Change in Host Populations

**Figure 2:**
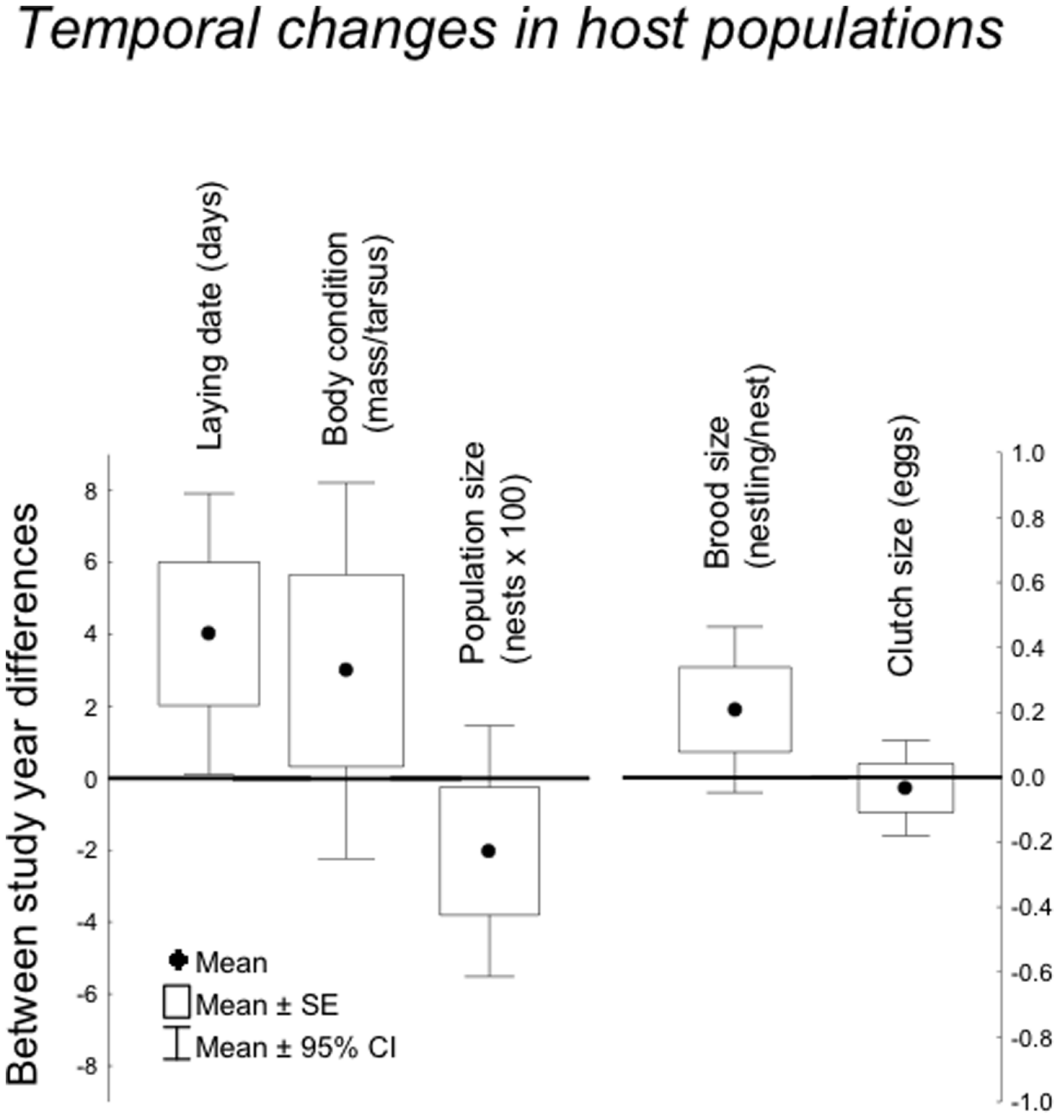
Temporal change in laying date of hosts, body condition of hosts, population size of hosts, brood size of hosts and clutch size of hosts between the first study year and 2010. Box plots show means, standard errors and 95% confidence intervals.

**Figure 3:**
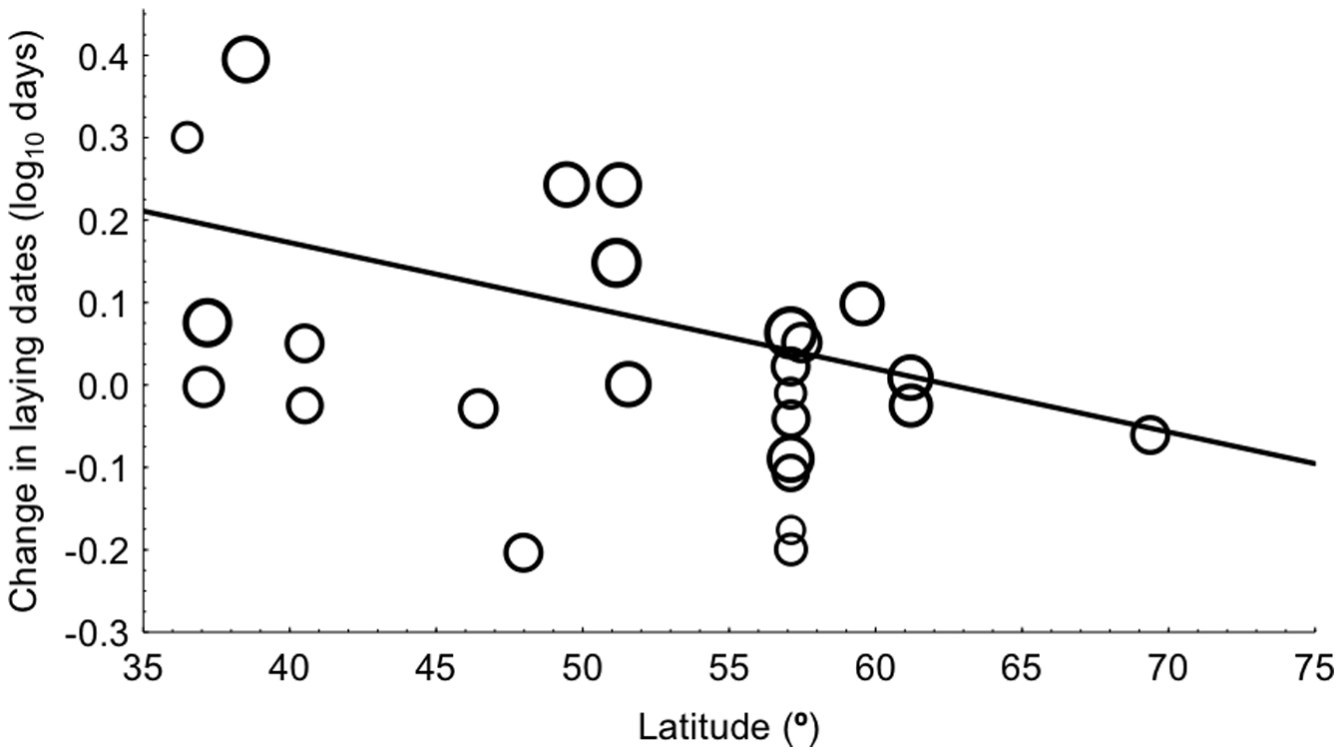
Change in laying date between first study year and 2010 in relation to latitude. The size of symbols is proportional to log-transformed sample size, while the lines are linear regression lines.

**Figure 4:**
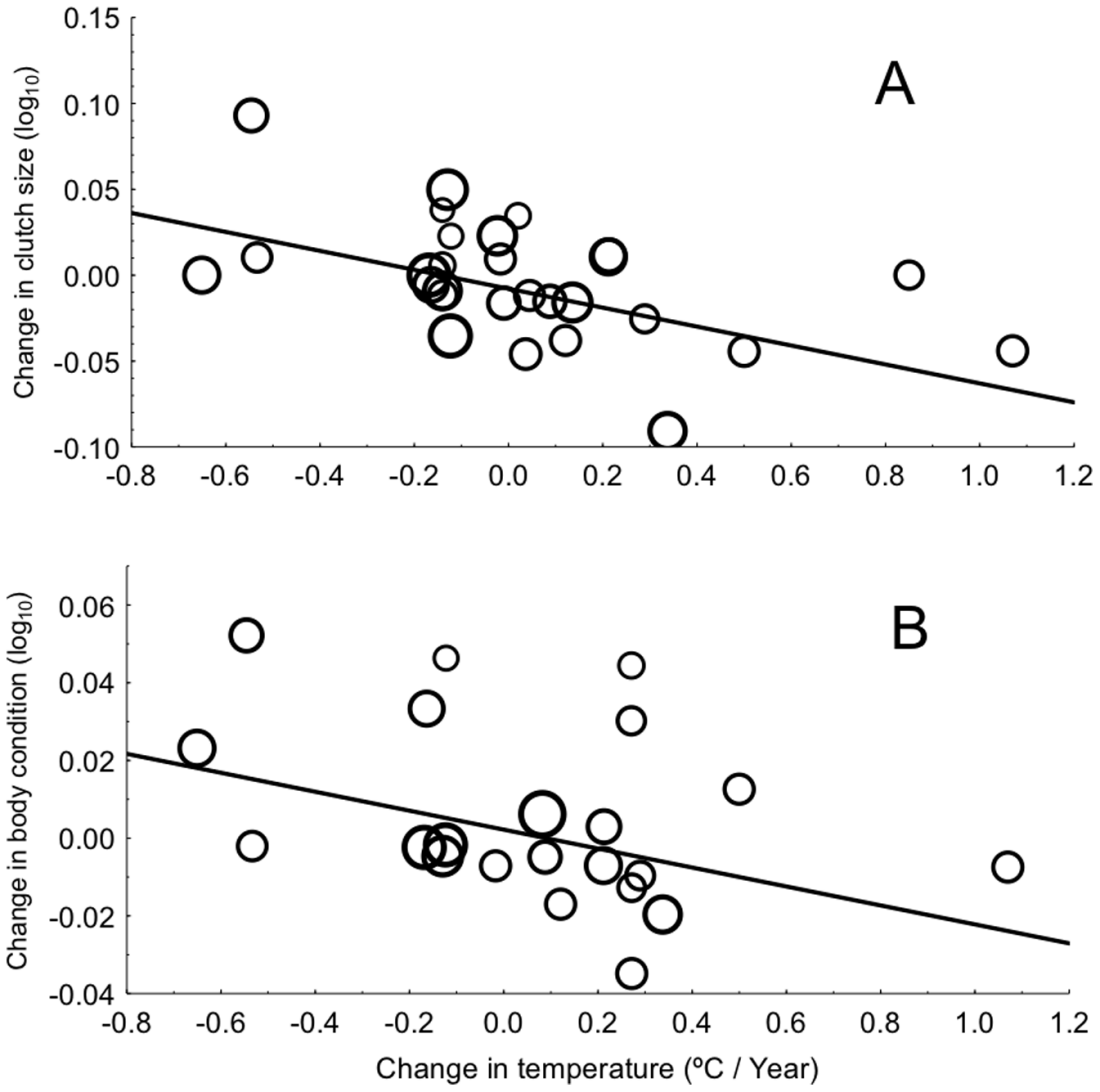
Host clutch size and body condition in relation to change in temperature. (A) Change in clutch size in relation to change in temperature. (B) Change in body condition in relation to change in temperature. The size of symbols is proportional to log-transformed sample sizes, while the lines are linear regression lines.

Host populations on average started to reproduce later during 2010 than during the first study year (Fig. 2). Moreover, temporal change in laying date differed significantly among localities (Table 1), which may be related to differential effects of climatic change at different latitudes (Table 1, Fig. 3). Change in clutch size decreased with increasing change in temperature (Table 1; Fig. 4A). Change in brood size differed among localities (Table 1), but not when considering studies with intervals between 5–15 years (Table S4 in File S1). Change in body condition tended to decrease with increasing temperature (Table 1; Fig. 4B). Host populations were less abundant in 2010 and this did not depend on host identity when studies with intervals between 5–15 years were considered (Table 1, Table S4 in File S1).

**Table 1:** Repeated measures ANOVAs with laying date, clutch size, brood size, body condition and population density of hosts in two separate study years as within subjects factors and locality identity, host identity, latitude, interval in years and temperature change (°C/year) as between subjects factors.

	Repeated measure			Locality			Host identity			Latitude			Interval in years			Temperature change (°C/year)		
	*F*	d.f.	*P*	*F*	d.f.	*P*	*F*	d.f.	*P*	*F*	d.f.	*P*	*F*	d.f.	*P*	*F*	d.f.	*P*
Laying date	**8.69**	**1,26**	**0.007**	**7.38**	**19,7**	**0.006**	1.17	15,11	0.406	**5.78**	**1,25**	**0.024**	0.02	1,25	0.900	2.49	1,25	0.127
Clutch size	0.86	1,29	0.363	0.83	20,9	0.654	0.80	17,12	0.673	0.27	1,28	0.606	0.27	1,28	0.606	**6.84**	**1,28**	**0.014**
Brood size	0.18	1,28	0.673	**3.53**	**19,9**	**0.029**	0.42	16,12	0.948	3.02	1,27	0.093	0.00	1,27	0.973	0.00	1,27	0.983
Body condition	0.57	1,22	0.458	1.00	12,10	0.505	1.22	14,8	0.400	0.14	1,21	0.710	0.18	1,21	0.675	**5.33**	**1,21**	**0.031**
Population density	**22.88**	**1,38**	**0.0001**	1.54	22,16	0.190	**5.51**	**22,16**	**0.001**	0.41	1,37	0.525	0.01	1,37	0.926	0.39	1,37	0.537

Each effect was estimated in separates models. P-values smaller than 0.05 are shown in bold.

### Temporal Change in Parasite Populations

**Figure 5:**
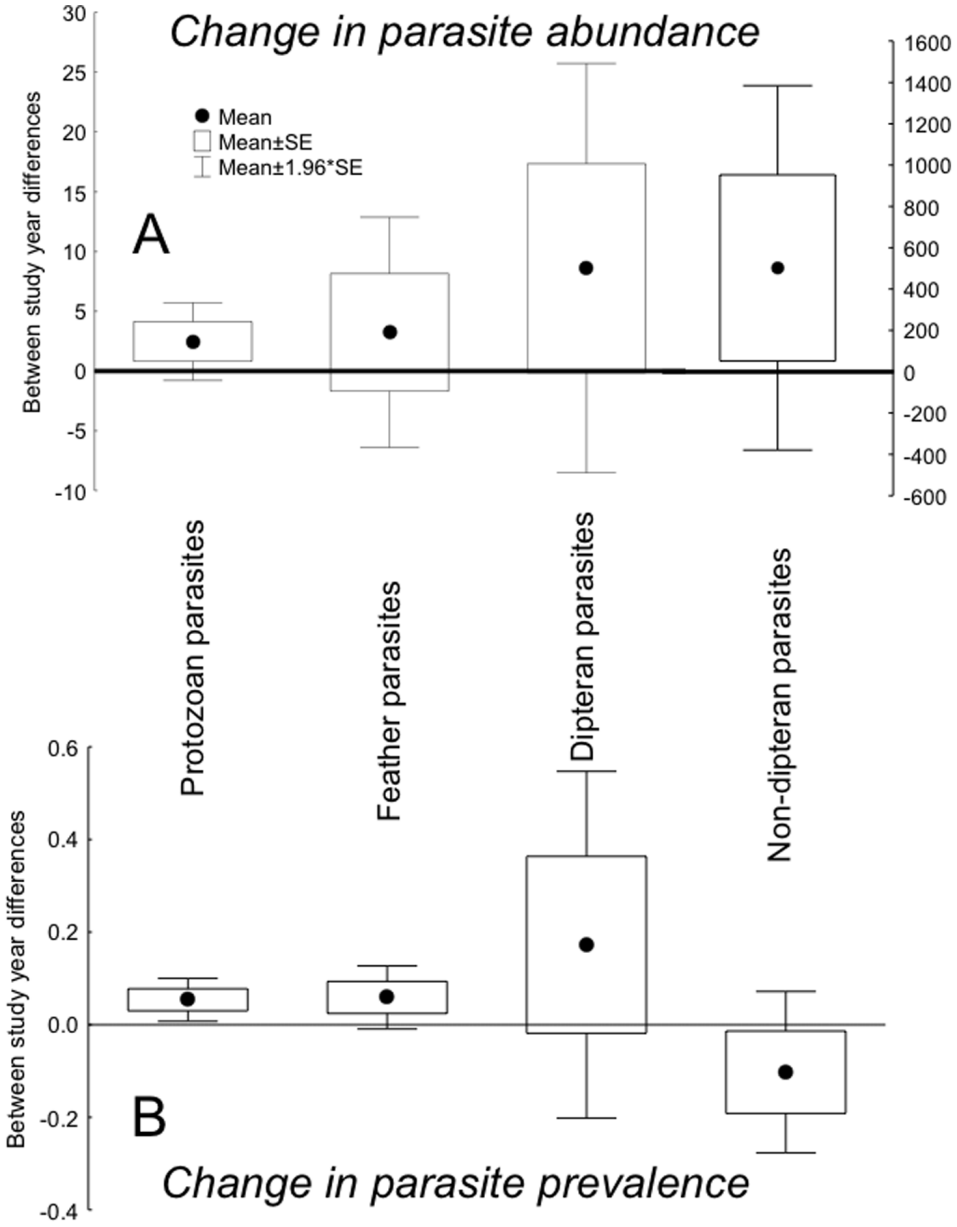
Change in parasite abundance and parasite prevalence over time for different categories of parasites. (A) Change in parasite abundance and (B) change in parasite prevalence between study years for different parasite taxa. Box plots show means, standard errors and 95% confidence intervals. The right Y-axis in (A) is the number of non-dipteran parasites.

We detected 18 gains and 8 losses of parasites among studies, and for studies with 5–15 years intervals there were 10 gains and 10 losses. Abundance of parasites was larger in 2010 than in the first study year. However, none of the variables explained change in abundance of parasites (Table 2; Fig. 5A). Variables that explained change in parasite prevalence depended on interval between study years. Locality was associated with parasite prevalence when considering studies with 5–15 years intervals (Table S5 in File S1), while parasite identity did so when all studies were considered (Table 2). Non-dipteran parasites were the only group that tended to decrease in prevalence between sampling years although this category did not differ significantly from tendencies for other categories (Table 2; Fig. 5B).

**Table 2:** Repeated measures ANOVAs with parasite abundance and prevalence in two separate study years as within subjects factors and locality identity, parasite identity, latitude, interval in years and temperature change (°C/year) as between subjects factors.

	Repeated measure			Locality			Parasite identity			Latitude			Interval in years			Temperature change (°C/year)		
	*F*	d.f.	*P*	*F*	d.f.	*P*	*F*	d.f.	*P*	*F*	d.f.	*P*	*F*	d.f.	*P*	*F*	d.f.	*P*
Parasite load	1.15	1,29	0.293	0.81	17,12	0.660	2.81	3,26	0.059	0.25	1,28	0.618	0.61	1,28	0.590	0.30	1,28	0.590
Parasite prevalence	0.19	1,43	0.668	0.89	20,23	0.597	**4.37**	**3,40**	**0.009**	0.51	1,18	0.943	0.01	1,18	0.943	0.187	1,18	0.668

Each effect was estimated in separates models. P-values smaller than 0.05 are shown in bold.

### Relationships between Temporal Change in Host and Parasite Populations

When considering the entire dataset, change in parasite abundance, but not in prevalence was associated with delayed laying (beta (SE) = 0.70 (0.21), *P* = 0.004) and reduced body condition (beta (SE) = −0.49 (0.16), *P* = 0.038) by their avian hosts (Table 3). Moreover, the rate of change in body condition and laying date of hosts depended on parasite group (Table 3). These associations were detected after controlling for the effects of latitude and temperature change (Table 3).

**Table 3:** Repeated measures ANOVAs and within subjects effects (parasite abundance/prevalence in the first and the second year) with laying date, clutch size, brood size, body condition and population density of hosts in two separate study years as within subjects factors and interactions between the repeated measure and latitude, temperature change, variation in parasitism (i.e. change in abundance and prevalence of parasites) and identity of parasite group as between subjects factors. P-values smaller than 0.05 are shown in bold.

	Repeated measure			Repeated measure * Latitude			Repeated measure * Temperature change			Repeated measure * Parasitism			Repeated measure * Parasite group		
	*F*	d.f.	*P*	*F*	d.f.	*P*	*F*	d.f.	*P*	*F*	d.f.	*P*	*F*	d.f.	*P*
Parasite abundance															
Laying date	**18.37**	**1,19**	**0.001**	**16.32**	**1,19**	**0.001**	2.95	1,19	0.102	**10.73**	**1,19**	**0.004**	**7.24**	**3,19**	**0.002**
Clutch size	0.67	1,20	0.422	0.07	1,20	0.456	1.27	1,20	0.274	3.77	1,20	0.066	1.57	3,20	0.227
Brood size	0.13	1,19	0.723	0.04	1,19	0.849	0.06	1,19	0.816	2.68	1,19	0.118	0.91	3,19	0.456
Body condition	**5.65**	**1,13**	**0.033**	**7.18**	**1,13**	**0.019**	**10.74**	**1,13**	**0.006**	**6.86**	**1,13**	**0.021**	**3.67**	**3,13**	**0.041**
Population density	**9.38**	**1,20**	**0.006**	**12.36**	**1,20**	**0.002**	0.01	1,20	0.924	0.93	1,20	0.347	1.94	3,20	0.156
Parasite prevalence															
Laying date	**16.47**	**1,25**	**0.001**	**5.05**	**1,25**	**0.034**	3.30	1,25	0.081	1.75	1,25	0.197	1.10	3,25	0.367
Clutch size	1.05	1,28	0.314	0.09	1,28	0.769	0.82	1,28	0.372	1.98	1,28	0.171	0.35	3,28	0.980
Brood size	0.73	1,28	0.401	0.01	1,28	0.912	0.06	1,28	0.801	1.84	1,28	0.186	0.27	3,28	0.844
Body condition	3.34	1,16	0.086	0.57	1,16	0.461	**5.72**	**1,16**	**0.029**	0.75	1,16	0.401	0.47	3,16	0.710
Population density	**5.69**	**1,28**	**0.024**	**10.19**	**1,28**	**0.003**	0.07	1,28	0.791	0.04	1,28	0.849	1.88	3,28	0.156

Interactions are indicated by *.

When only considering studies with an interval of 5–15 years, the association between laying date and parasite abundance did not reach statistical significance, but change in laying date differed significantly among parasite categories when either parasite abundance or parasite prevalence were included in the model (Table S6 in File S1). Interestingly, these restricted analyses showed not only the detected negative association between change in parasite abundance and change in body condition of hosts (beta (SE) = −0.49 (0.16), *P* = 0.038), but also significant effects of parasite abundance on clutch size (beta (SE) = −0.61 (0.23), *P* = 0.026) and brood size (beta (SE) = −0.58 (0.21), *P* = 0.021) (Table S6 in File S1). Finally, when considering this restricted sample of studies, the results indicate that parasite categories explained changes in population density of hosts when parasite abundance was included in the model (Table S6 in File S1). All these associations were detected after controlling for the effects of latitude and temperature change (Table S6 in File S1).

## Discussion

We posed the question whether a general increase in temperature had a significant effect on abundance of parasites and hosts, and whether this led to altered effects of parasites on host fitness. We recorded 18 gains and 8 losses of parasites although a more balanced frequency of 10 gains and 10 losses were found for the restricted dataset. Hosts generally started to reproduce later in recent years, and we could link this change in clutch size and change in body condition to change in temperature, although obviously other unknown factors could have changed as well. Change in parasite abundance and prevalence was not significantly related to change in temperature. Change in parasite abundance, but not change in parasite prevalence, explained a significant proportion of variance in host life-history variables. Specifically, increasing parasite abundance was associated with clutch size, brood size and body condition, as was the increase in temperature. This suggests that changes in parasite abundance occurred through indirect effects such as effects of temperature on hosts. This raises the possibility that other aspects of climate change such as precipitation or wind could affect insect vectors and larval nematodes. Finally, population size of hosts was not significantly correlated with abundance and prevalence of parasites or change in temperature.

Change in abundance of parasites was linked to change in temperature during the breeding season of hosts, which in general is also the breeding season of the parasites. This general finding is in accordance with several studies that have documented changes in prevalence and/or abundance of parasites over time [34], or in response to change in climate [33], [35], [36]. We caution that change in parasite prevalence or abundance over time may be linked to many factors other than climate change. Long-term studies of the same population of hosts and parasites during decades in areas where temperature has increased dramatically are clearly more powerful, and hence more likely to find predicted changes [33] than studies based on two years.

Increasing temperatures advance the emergence of resting stages of parasites [4], [23]–[25], [33], [48] or their vectors [33], [34], [49]. This can affect the fitness costs of parasitism for hosts because parasites inflict greater fitness costs when weather is poor [50]–[52], and earlier start of reproduction by hosts is likely to increase the risk of adverse weather. Likewise higher temperatures can increase reproductive rates of parasites and thus the rate of evolution by parasites relative to that of hosts [4], [23], [24], [48], even when hosts also increase their reproductive rate [33]. Finally, climate may affect defenses of hosts and thereby increase the level of virulence of parasites [4], [13], [37], [53], [54]. These scenarios predict changes in parasite populations associated with climate change as shown by our results when restricting the analyses to studies with an interval of 5–15 years. In addition, we found support for the prediction that temperature explained change in clutch size and body condition of hosts, and both these fitness components were associated with parasite abundance. This provides evidence of an indirect effect of parasites on fitness components of hosts associated with an effect of temperature on the same fitness components.

The support for our predictions when restricting the analyses to studies with intervals of 5–15 years suggests that a certain amount of time must have passed to result in changes. We found associations between change in temperature and change in clutch size and change in body condition, respectively, although similar effects were absent for laying date and brood size. Factors such as those related to host characteristics, and whether parasites spend part of the year away from hosts, may be important.

We found an increase in abundance of parasites in 2010 compared to the first year when restricting the analyses to datasets with an interval of 5–15 years. There was no confounding effect of population density of hosts because host population density on average decreased during the course of the study, thereby reducing parasite transmission rates. Most of the parasite species were endoparasites living permanently within their hosts, hence only indirectly being affected by changing temperatures. We can only speculate that the detected change in the abundance of parasites for populations may be related to the indirect effects of temperature on host population density (Results) and/or an increase in immunity and anti-parasite responses of hosts [14], [55], [56].

The parasite species have all at least sometimes been shown to negatively impact fitness components of their hosts [8]. We detected that species, which experienced an increase in the abundance of parasites during the study period, advanced their laying date compared to species in which parasite abundance decreased. However, this effect disappeared when considering the restricted dataset that only included studies with an interval of 5–15 years. With this restricted dataset we found evidence of change in body condition, clutch size and brood size of hosts being negatively linked to change in temperature and parasite abundance, while changes in body condition and clutch size were also associated with change in temperature. These associations were not detected when considering parasite prevalence. While parasite abundance reflects the impact of parasites on hosts at the level of individual hosts, prevalence constitutes a measure of parasite populations at the level of host populations. Our results are therefore in accordance with the assumption that parasitism negatively influences reproductive success of their hosts, and with the hypothesis that the effect of climate change on host reproduction is at least partially mediated by changes in parasitism due to climate change.

While changes in the distribution and abundance of parasites may impact their hosts, whether this has any population consequence for the host depends on density-dependent mortality and fecundity [57]–[59]. Populations which have declined the most are those that have not responded to climate change [9], [35], [60], [61]. We found no evidence for an effect of climate change on population size of birds as mediated by parasites. Change in population density of hosts depended on latitude, and it differed among parasite groups. These findings suggest that climate change has not affected population size of hosts directly through effects of change in parasitism or change in temperature.

In conclusion, while climate change may affect hosts by affecting phenology, reproduction and host body condition, an increase in temperature was associated with a general increase in parasite abundance. Clutch size, brood size and body condition of hosts were associated with a change in temperature and with a change in parasitism, respectively, suggesting that parasites may affect fecundity and condition of their hosts.

## Supporting Information

File S1 **Data files and supplementary analyses of determinants of parasitism and host life history.** Table S1. Summary information on parasitism and study populations. Table S2. Summary information on study populations and intensity and prevalence of parasitism. Table S3. Correlations between parasitism and life history traits. Table S4. Analyses of host populations based on data with an interval of sampling of 5–15 years. Table S5. Analyses of determinants of parasitism based on data with an interval of sampling of 5–15 years. Table S6. Analyses of parasitism in relation to life history based on data with an interval of sampling of 5–15 years.
(DOC)

## References

[1] Price PW (1980) Evolutionary biology of parasites. Princeton: Princeton University Press.

[2] Bush AO, Fernández JC, Esch GW, Seed JR (2001) Parasitism. The diversity and ecology of animal parasites. Cambridge: Cambridge University Press.

[3] Combes C (2001) Parasitism: The ecology and evolution of intimate interactions. Chicago: University of Chicago Press.

[4] Merino S, Møller AP (2010) Host-parasite interactions and climate change. In: Møller AP, Fiedler W, Berthold P (eds) Birds and climate change. Oxford: Oxford University Press, pp. 213–226.

[5] Guérnier V, Hochberg ME, Guégan J-F (2004) Ecology drives the worldwide distribution of human diseases. PLoS Biol 2: 740–746. 10.1371/journal.pbio.0020141 PMC423130 15208708

[6] Merino S, Moreno J, Vásquez RA, Martínez J, Sánchez-Monsálvez I, et al (2008) Haematozoa in forest birds from southern Chile: Latitudinal gradients in prevalence and parasite lineage richness. Austral Ecol 33: 329–340.

[7] Rohde K (1998) Latitudinal gradients in species diversity. Area matters, but how much? Oikos 82: 184–190.

[8] Møller AP, Arriero E, Lobato E, Merino S (2009) A meta-analysis of parasite virulence in nestling birds. Biol Rev 84: 567–588. 19673856 10.1111/j.1469-185X.2009.00087.x

[9] Guégan J-F, Thomas F, Hochberg ME, de Meeus T, Renaud F (2003) Disease diversity and human fertility. Evolution 55: 1308–1314. 10.1111/j.0014-3820.2001.tb00653.x 11525455

[10] Guégan J-F, Morand S, Poulin R (2005)) Are there general laws in parasite community ecology? The emergence of spatial parasitology and epidemiology. In: Thomas F, Renaud F, Guégan J-F (eds) Parasitism and ecosystems. Oxford: Oxford University Press, pp. 22–42.

[11] Barbosa A, Merino S, Benzal J, Martinez J, Garcia-Fraile S (2007) Geographic variation in the immunoglobulin levels in pygoscelid penguins. Polar Biol 30: 219–225.

[12] Møller AP (1998) Evidence of larger impact of parasites on hosts in the tropics: Investment in immune function within and outside the tropics. Oikos 82: 265–270.

[13] Møller AP, Martín-Vivaldi M, Merino S, Soler JJ (2006) Density-dependent and geographical variation in bird immune response. Oikos 115: 463–474.

[14] Martínez J, Merino S (2011) Host- parasite interactions under extreme climatic conditions. Curr Zool 57: 390–405.

[15] Brooks DR, Hoberg EP (2007) How will global climate change affect parasite–host assemblages? Trends Parasitol 23: 571–574. 17962073 10.1016/j.pt.2007.08.016

[16] de la Rocque S, Rioux JA, Slingenbergh J (2008) Climate change: effects on animal disease systems and implications for surveillance and control. Rev sci technique, Office int épizoo 27: 339–354. 18819664

[17] Dobson A (2009) Climate variability, global change, immunity, and the dynamics of infectious diseases. Ecology 90: 920–927. 19449686 10.1890/08-0736.1

[18] Gilbert M, Slingenbergh J, Xiao X (2008) Climate change and avian influenza. Rev sci technique, Office int épizoo 27: 459–466. PMC2709837 18819672

[19] Harvell D, Altizer S, Cattadori IM, Harrington L, Weil E (2009) Climate change and wildlife diseases: When does the host matter the most? Ecology 90: 912–930. 19449685 10.1890/08-0616.1

[20] Lafferty KD (2009) The ecology of climate change and infectious diseases. Ecology 90: 888–900. 19449681 10.1890/08-0079.1

[21] Lovejoy T (2008) Climate change and biodiversity. Rev sci technique, Office int épizoo 27: 331–338. 18819663

[22] Mas-Coma S, Valero MA, Bargues MD (2008) Effects of climate change on animal and zoonotic helminthiases. Rev sci technique, Office int épizoo 27: 443–452. 18819671

[23] Poulin R (2006) Global warming and temperature-mediated increases in cercarial emergence in trematode parasites. Parasitol 132: 143–151. 10.1017/S0031182005008693 16393363

[24] Poulin R, Mouritsen KN (2006) Climate change, parasitism and the structure of intertidal ecosystems. J Helminthol 80: 183–191. 16768861 10.1079/joh2006341

[25] Calero-Torralbo MA, Václav R, Valera F (2013) Intra-specific variability in life-cycles synchronization between an ectoparasitic fly and its avian host. Oikos 122: 274–284.

[26] Ogden NH, Maarouf A, Barker IK, Bigras-Poulin M, Lindsay LR, et al (2006) Climate change and the potential for range expansion of the Lyme disease vector *Ixodes scapularis* in Canada. Int J Parasitol 36: 63–70. 16229849 10.1016/j.ijpara.2005.08.016

[27] Cadenas FM, Rais O, Jouda F, Douet V, Humair P-F, et al (2007) Phenology of *Ixodes ricinus* and infection with *Borrelia burgdorferi* sensu lato along a North- and South-facing altitudinal gradient on Chaumont Mountain, Switzerland. J Med Entomol 44: 683–693. 17695026 10.1603/0022-2585(2007)44[683:poirai]2.0.co;2

[28] Dunn PO, Winkler DW (2010) Effects of climate change on timing of breeding and reproductive success in birds. In: Møller AP, Fiedler W, Berthold P (eds) Effects of climate change on birds. Oxford: Oxford University Press, pp. 113–128.

[29] Møller AP, Flensted-Jensen E, Klarborg K, Mardal W, Nielsen JT (2010) Climate change affects the duration of the reproductive season in birds. J Anim Ecol 79: 777–784. 20202013 10.1111/j.1365-2656.2010.01677.x

[30] Møller AP (2002) North Atlantic Oscillation (NAO) effects of climate on the relative importance of first and second clutches in a migratory passerine birds. J Anim Ecol 71: 201–210.

[31] Møller AP (2007) Interval between clutches, fitness and climate change. Behav Ecol 18: 62–70.

[32] Møller AP, Cassey P (2004) On the relationship between T-cell mediated immunity in bird species and the establishment success of introduced populations. J Anim Ecol 73: 1035–1042.

[33] Møller AP (2010) Host-parasite interactions and vectors in the barn swallow in relation to climate change. Global Change Biol 16: 1158–1170.

[34] Garamszegi LZ (2011) Climate change increases the risk of malaria in birds. Global Change Biol 17: 1751–1759.

[35] Saino N, Rubolini D, Lehikoinen E, Sokolov LV, Bonisoli-Alquati A, et al (2009) Climate change effects on migration phenology may mismatch brood parasitic cuckoos and their hosts. Biol Lett 5: 539–541. 19443508 10.1098/rsbl.2009.0312 PMC2781939

[36] Møller AP, Saino N, Adamík P, Ambrosini R, Antonov A, et al (2011) Rapid change in host use of the common cuckoo *Cuculus canorus* linked to climate change. Proc R Soc Lond B 278: 733–738. 10.1098/rspb.2010.1592 PMC3030850 20843848

[37] Møller AP, Erritzøe J (2003) Climate, body condition and spleen size in birds. Oecologia 137: 621–626. 13680351 10.1007/s00442-003-1378-1

[38] Sokal RR, Rohlf FJ (2005) Biometry. New York: Freeman.

[39] Fichet-Calvet E, Jomaa I, Ben Ismail R, Ashford RW (2003) *Leishmania major* infection in the fat sand rat *Psammomys obesus* in Tunisia: interaction of host and parasite populations. Ann Trop Med Parasitol 97: 593–603. 14511558 10.1179/000349803225001517

[40] Khan R (2008) Influence of environmental changes in the north-western Atlantic Ocean on a parasite, *Echinorhynchus gadi* (Acanthocephala) of Atlantic cod (*Gadus morhua*) occurring off coastal Labrador, Canada. J Helminthol 82: 203–209. 18452629 10.1017/S0022149X0897615X

[41] Khan RA, Chandra CV (2006) Influence of climatic changes on the parasites of Atlantic cod *Gadus morhua* off coastal Labrador, Canada. J Helminthol 80: 193–197. 16768862 10.1079/joh2006352

[42] Morley N, Lewis J (2008) The influence of climatic conditions on long-term changes in the helminth fauna of terrestrial molluscs and the implications for parasite transmission in southern England. J Helminthol 82: 325–335. 18598579 10.1017/S0022149X0802645X

[43] Merino S, Potti J, Fargallo JA (1997) Blood parasites of some passerine birds from central Spain. J Wildl Dis 33: 638–641. 9249714 10.7589/0090-3558-33.3.638

[44] Haylock MR, Hofstra N, Klein Tank AMG, Klok EJ, Jones PD, et al (2008) A European daily high-resolution gridded dataset of surface temperature and precipitation. J Geophys Res (Atmospheres) 116: D11110.

[45] Garamszegi LZ, Møller AP (2010) Effects of sample size and intraspecific variation in phylogenetic comparative studies: A meta-analytic review. Biol Rev 85: 797–805. 20148861 10.1111/j.1469-185X.2010.00126.x

[46] Garamszegi LZ, Møller AP (2011) Non-random variation in within-species sample size and missing data in phylogenetic comparative studies. Syst Biol 60: 876–880. 21712480 10.1093/sysbio/syr060

[47] Statsoft Inc (2011) STATISTICA (data analysis software system), version 10. www.statsoft.com.

[48] Dawson RD, Hillen KK, Whitworth TL (2005) Effects of experimental variation in temperature on larval densities of parasitic *Protocalliphora* (Diptera: Calliphoridae) in nests of tree swallows (Passeriformes: Hirundinidae). Environ Entomol 34: 563–568.

[49] Martínez-de la Puente J, Merino S, Lobato E, et al (2009) Does weather affect biting fly abundance in avian nests? J Avian Biol 40: 653–657.

[50] de Lope F, Møller AP, González G, Peréz JJ (1993) Increased detrimental effects of ectoparasites on their bird hosts during adverse environmental conditions. Oecologia 95: 234–240. 28312947 10.1007/BF00323495

[51] Dufva R, Allander K (1996) Variable effects of the hen flea *Ceratophyllus gallinae* on the breeding success of the great tit *Parus major* in relation to weather conditions. Ibis 138: 772–777.

[52] Merino S, Potti J (1996) Weather dependent effects of ectoparasites on their bird host. Ecography 19: 107–113.

[53] Plischke A, Quillfeldt P, Lubjuhn T, Merino S, Masello JF (2009) Leucocytes in adult burrowing parrots *Cyanoliseus patagonus* in the wild: Variation between contrasting breeding seasons, genders and individual condition. J Ornithol 151: 347–354.

[54] Guirguis K, Gershunov A, Schwartz R, Bennett S (2011) Recent warm and cold daily winter temperature extremes in the Northern Hemisphere. Geophys Res Lett 38: L17701.

[55] Sinclair JA, Lochmiller RL (2000) The winter immunoenhancement hypothesis: associations among immunity, density, and survival in prairie vole (*Microtus ochrogaster*) populations. Can J Zool 78: 254–264.

[56] Zahraa HA (2008) Effects of commutative heat stress on immunoresponses in broiler chickens reared in closed system. Int J Poultry Sci 7: 964–968.

[57] Hudson PJ, Cattadori IM, Boag B, Dobson AP (2006) Climate disruption and parasite-host dynamics: patterns and processes associated with warming and the frequency of extreme climatic events. J Helminthol 80: 175–182. 16768860 10.1079/joh2006357

[58] Cattadori IM, Haydon DT, Hudson PJ (2005) Parasites and climate synchronize red grouse populations. Nature 433: 737–741. 15716952 10.1038/nature03276

[59] Møller AP (2005) Parasitism and the regulation of host populations. In: F Thomas, F Renaud, J-F Guégan (eds) Parasitism and ecosystems. Oxford: Oxford University Press, pp. 43–53.

[60] Both C, Bouwhuis S, Lessells CM, Visser ME (2006) Climate change and population declines in a long distance migratory bird. Nature 441: 81–83. 16672969 10.1038/nature04539

[61] Both C, Van Turnhout CAM, Bijlsma RG, Siepel H, Van Strien AJ, et al (2010) Avian population consequences of climate change are most severe for long-distance migrants in seasonal environments. Proc R Soc B 277: 1259–1266. 10.1098/rspb.2009.1525 PMC2842804 20018784

