# Supplementary material for: Assessing the Effects of Climate on Host-Parasite Interactions: A Comparative Study of European Birds and Their Parasites
Source: PLoS One. 2013 Dec 31;8(12):e82886. doi: 10.1371/journal.pone.0082886 (PMC3876993; doi:10.1371/journal.pone.0082886)
Supplement: File S1 — Data files and supplementary analyses of determinants of parasitism and host life history. Table S1. Summary information on parasitism and study populations. Table S2. Summary information on study populations and intensity and prevalence of parasitism. Table S3. Correlations between parasitism and life history traits. Table S4. Analyses of host populations based on data with an interval of sampling of 5–15 years. Table S5. Analyses of determinants of parasitism based on data with an interval of sampling of 5–15 years. Table S6. Analyses of parasitism in relation to life history based on data with an interval of sampling of 5–15 years. (DOC) [file pone.0082886.s001.doc]

File S1. Data files and supplementary analyses of determinants of parasitism and host life history.

Supplementary Table S1. Summary information on parasitism and study populations. Summary information on parasite and host taxa, whether the parasites are ecto- or endo-parasites, population size of hosts in year 1 and year 2, year 1 and year 2, study site, change in temperature during the main month of reproduction (ºC/yr), and the person(s) responsible for collecting the data.

| Parasite species | Parasite taxon | Ectoparasite | Endoparasite | Host species | Population size of host in year 1 | Population size of host in year 2 | Year 1 | Year 2 | Study site | Change in temperature (ºC/yr) | Data collector |
| --- | --- | --- | --- | --- | --- | --- | --- | --- | --- | --- | --- |
| *Trichomonas gallina*e | Bacteria | 0 | 1 | *Accipiter gentilis* | 31 | 37 | 1977 | 2010 | Sindal | 0.066 | J. T. Nielsen |
| Mite | Mite | 1 | 0 | *Acrocephalus scirpaceus* | 23 | 18 | 2005 | 2010 | Kraghede | 0.052 | A. P. Møller |
| *Trichomonas gallinae* | Bacteria | 0 | 1 | *Columba oenas* | 45 | 45 | 2006 | 2010 | Frederikshavn | -0.218 | E. Flensted-Jensen |
| Mite | Mite | 1 | 0 | *Cyanistes caeruleus* | 6.5 | 6 | 2001 | 2010 | Algeria | -0.129 | N. Ziane |
| Ticks | Ticks | 1 | 0 | *Cyanistes caeruleus* | 6.5 | 6 | 2001 | 2010 | Algeria | -0.129 | N. Ziane |
| *Dermanyssus* sp. | Mite | 1 | 0 | *Cyanistes caeruleus* | 61 | 72 | 2005 | 2010 | Valsaín | 0.002 | J. Rivero de Aguilar & S. Merino |
| *Protocalliphora azurea* | Blowfly | 1 | 0 | *Cyanistes caeruleus* | 61 | 72 | 2005 | 2010 | Valsaín | 0.002 | J. Rivero de Aguilar & S. Merino |
| *Ceratophyllus gallinae* | Flea | 1 | 0 | *Cyanistes caeruleus* | 61 | 72 | 2005 | 2010 | Valsaín | 0.002 | J. Rivero de Aguilar & S. Merino |
| *Culicoides* sp. | Biting fly | 1 | 0 | *Cyanistes caeruleus* | 61 | 72 | 2005 | 2010 | Valsaín | 0.002 | J. Rivero de Aguilar, J. Martínez-de la Puente & S. Merino |
| Chewing louse | Chewing louse | 1 | 1 | *Delichon urbica* | 636 | 468 | 2000 | 2010 | Badajoz | 0.146 | A. Marzal |
| Feather mite | Feather mites | 1 | 1 | *Delichon urbica* | 456 | 468 | 2003 | 2010 | Badajoz | 0.082 | A. Marzal |
| Louse fly | Lousefly | 1 | 0 | *Delichon urbica* | 32 | 16 | 1971 | 2010 | Kraghede | 0.050 | A. P. Møller |
| *Ceratophyllus gallinae* | Flea | 1 | 0 | *Falco tinnunculus* | 12 | 11 | 2005 | 2010 | Kraghede | 0.052 | A. P. Møller |
| *Ornithonyssus bursa* | Mite | 1 | 0 | *Hirundo rustica* | 261 | 124 | 1993 | 2010 | Kraghede | 0.109 | A. P. Møller |
| *Ornithomyia avicularia* | Lousefly | 1 | 0 | *Hirundo rustica* | 261 | 124 | 1993 | 2010 | Kraghede | 0.109 | A. P. Møller |
| *Brueelia* sp. | Chewing louse | 1 | 1 | *Hirundo rustica* | 261 | 124 | 1993 | 2010 | Kraghede | 0.109 | A. P. Møller |
| *Trouessartia crucifera* | Feather mite | 1 | 1 | *Hirundo rustica* | 261 | 124 | 1993 | 2010 | Kraghede | 0.109 | A. P. Møller |
| *Brueelia* sp. | Chewing louse | 1 | 1 | *Hirundo rustica* | 90 | 45 | 2000 | 2010 | Voronkov | 0.085 | A. P. Møller & T. A. Mousseau |
| *Brueelia* sp. | Chewing louse | 1 | 1 | *Hirundo rustica* | 147 | 66 | 2000 | 2010 | Chernobyl | 0.085 | A. P. Møller & T. A. Mousseau |
| *Trouessartia crucifera* | Feather mite | 1 | 1 | *Hirundo rustica* | 90 | 45 | 2000 | 2010 | Voronkov | 0.085 | A. P. Møller & T. A. Mousseau |
| *Trouessartia crucifera* | Feather mite | 1 | 1 | *Hirundo rustica* | 147 | 66 | 2000 | 2010 | Chernobyl | 0.085 | A. P. Møller & T. A. Mousseau |
| Chewing louse | Chewing louse | 1 | 1 | *Hirundo rustica* | 47 | 52 | 2007 | 2010 | Poland | -0.593 | P. Matyjasiak |
| Mite | Mite | 1 | 0 | *Hirundo rustica* | 11.6 | 20.4 | 1997 | 2010 | Cojocna | 0.174 | P. L. Pap |
| *Ceratophyllus gallinae* | Flea | 1 | 0 | *Parus major* | 84 | 106 | 2005 | 2010 | Rødhus | 0.052 | K. Klarborg |
| *Ixodes ricinus* | Ticks | 1 | 0 | *Parus major* | 178 | 154 | 1997 | 2010 | Antwerp | 0.152 | D. Heylen and E. Matthysen |
| *Protocalliphora azurea* | Blowfly | 1 | 0 | *Parus major* | 94 | 86 | 1991 | 2010 | Harjavalta | 0.086 | T. Eeva |
| *Ceratophyllus gallinae* | Flea | 1 | 0 | *Parus palustris* | 5 | 7 | 2005 | 2010 | Rødhus | 0.052 | K. Klarborg |
| Feather mites | Feather mite | 1 | 1 | *Passer domesticus* | 25 | 23 | 2007 | 2010 | Kraghede | -0.264 | A. P. Møller |
| *Carnus* sp. | Fly | 1 | 0 | *Pica pica* | 71 | 91 | 1997 | 2010 | Hoya de Guadix | 0.047 | J. Soler & M. Molina |
| Chewing louse | Chewing louse | 1 | 1 | *Riparia riparia* | 8588 | 1823 | 1992 | 2010 | Hungary | 0.022 | T. Szép |
| Ticks | Ticks | 1 | 0 | *Riparia riparia* | 8588 | 1823 | 1992 | 2010 | Hungary | 0.022 | T. Szép |
| *Dermanyssus gallinae* | Mite | 1 | 0 | *Sturnus vulgaris* | 62 | 88 | 1996 | 2010 | Southern Bavaria | 0.169 | H. Gwinner |
| *Ceratophyllus gallinae* | Flea | 1 | 0 | *Turdus merula* | 15 | 15 | 2005 | 2010 | Kraghede | 0.052 | A. P. Møller |

Supplementary Table S2. Summary information on study populations and intensity and prevalence of parasitism. Information on latitude (ºN), longitude (ºE), mean intensity of infection in year 1, SE of intensity of infection in year 1, prevalence in year 1, sample size in year 1, mean intensity of infection in year 2, SE of intensity of infection in year 2, prevalence in year 2, and sample size in year 2.

| Parasite species | Latitude | Longitude | Intensity year 1 | SE year 1 | Prevalence year 1 | N year 1 | Intensity year 2 | SE year 2 | Prevalence year 2 | N year 2 |
| --- | --- | --- | --- | --- | --- | --- | --- | --- | --- | --- |
| *Trichomonas gallina*e | 57.5 | 10.22 | 0.000 | 0.000 | 0.000 | 31 | . | . | 0.270 | 114 |
| Mite | 57.12 | 10 | . | . | 0.667 | 12 | . | . | 0.500 | 224 |
| *Trichomonas gallinae* | 57.26 | 10.32 | 0.000 | 0.000 | 0.000 | 14 | 0.222 | 0.428 | 0.101 | 224 |
| Mite | 36.51 | 8.19 | 156.125 | 21.277 | 1.000 | 8 | 3428.857 | 1302.152 | 1.000 | 224 |
| Ticks | 36.51 | 8.19 | 76.375 | 8.237 | 1.000 | 8 | 3165.000 | 973.865 | 1.000 | 54 |
| *Dermanyssus* sp. | 40.53 | -4.01 | 300.429 | 58.411 | 1.000 | 14 | 1059.429 | 329.318 | 0.952 | 69 |
| *Protocalliphora azurea* | 40.53 | -4.01 | 16.600 | 3.521 | 0.867 | 15 | 47.250 | 4.891 | 0.950 | 54 |
| *Ceratophyllus gallinae* | 40.53 | -4.01 | 76.714 | 38.100 | 0.500 | 14 | 103.952 | 65.024 | 0.238 | 69 |
| *Culicoides* sp. | 40.53 | -4.01 | 27.062 | 9.261 | 0.938 | 16 | 95.095 | 25.595 | 1.000 | 14 |
| Chewing louse | 38.5 | -6.59 | 11.893 | 1.434 | 0.976 | 84 | 67.266 | 2.337 | 0.970 | 17 |
| Feather mite | 38.5 | -6.59 | 17.381 | 1.331 | 0.824 | 176 | 9.378 | 0.407 | 0.978 | 6 |
| Louse fly | 57.12 | 10 | 0.000 | 0.000 | 0.000 | 32 | 8.000 | 1.600 | 0.750 | 11 |
| *Ceratophyllus gallinae* | 57.12 | 10 | 9.920 | 3.370 | 0.916 | 12 | 21.450 | 0.610 | 0.909 | 106 |
| *Ornithonyssus bursa* | 57.12 | 10 | 0.521 | 0.064 | 0.303 | 188 | 0.000 | 0.000 | 0.000 | 7 |
| *Ornithomyia avicularia* | 57.12 | 10 | 0.000 | 0.000 | 0.000 | 259 | 0.277 | 0.050 | 0.232 | 18 |
| *Brueelia* sp. | 57.12 | 10 | 13.080 | 0.790 | 0.772 | 257 | 9.640 | 0.640 | 0.768 | 37 |
| *Trouessartia crucifera* | 57.12 | 10 | 68.860 | 3.980 | 0.844 | 257 | 28.670 | 1.770 | 0.844 | 7 |
| *Brueelia* sp. | 49.45 | 31.28 | 14.000 | 1.670 | 0.935 | 92 | 10.040 | 1.240 | 0.796 | 7 |
| *Brueelia* sp. | 51.23 | 30.05 | 13.740 | 1.370 | 0.800 | 55 | 9.770 | 7.910 | 0.812 | 115 |
| *Trouessartia crucifera* | 49.45 | 31.28 | 34.140 | 11.100 | 0.674 | 92 | 35.460 | 3.820 | 0.926 | 51 |
| *Trouessartia crucifera* | 51.23 | 30.05 | 20.800 | 3.220 | 0.655 | 55 | 63.860 | 5.020 | 0.986 | 24 |
| Chewing louse | 51.57 | 20.05 | 21.990 | 1.790 | 0.904 | 93 | 26.275 | 2.382 | 0.961 | 369 |
| Mite | 46.44 | 23.5 | 58.200 | 18.816 | 0.560 | 25 | 1.759 | 1.723 | 0.069 | 29 |
| *Ceratophyllus gallinae* | 57.12 | 9.4 | 0.870 | 0.340 | 0.131 | 84 | 2.120 | 0.670 | 0.207 | 504 |
| *Ixodes ricinus* | 51.16 | 4.3 | 0.200 | 0.070 | 0.160 | 50 | 2.226 | 0.305 | 0.652 | 2736 |
| *Protocalliphora azurea* | 61.2 | 22.1 | 9.722 | 2.009 | 0.611 | 72 | 2.882 | 1.197 | 0.240 | 76 |
| *Ceratophyllus gallinae* | 57.12 | 9.4 | 0.000 | 0.000 | 0.000 | 5 | 2.430 | 2.110 | 0.286 | 75 |
| Feather mites | 57.12 | 10 | 0.000 | 0.000 | 0.000 | 28 | 0.714 | 0.412 | 0.214 | 16 |
| *Carnus* sp. | 37.18 | -3.11 | 1.747 | 0.184 | 0.800 | 40 | 1.065 | 0.163 | 0.710 | 28 |
| Chewing louse | 48.12 | 21.47 | 1.643 | 0.163 | 0.445 | 272 | 2.419 | 0.205 | 0.437 | 21 |
| Ticks | 48.12 | 21.47 | 0.054 | 0.005 | 0.042 | 3952 | 0.004 | 0.002 | 0.003 | 20 |
| Mite | 48 | 11 | 1.083 | 0.058 | . | 24 | 1.375 | 0.101 | . | 21 |
| *Ceratophyllus gallinae* | 57.12 | 10 | 0.850 | 0.420 | 0.400 | 20 | 2.400 | 0.990 | 0.240 | 21 |

Supplementary Table S3. Correlations between parasitism and life history traits. Pearson product-moment correlation coefficient between intensity of infection and laying date in year 1, correlation coefficient between intensity of infection and clutch size in year 1, correlation coefficient between intensity of infection and reproductive success in year 1, correlation coefficient between intensity of infection and body condition in year 1, correlation coefficient between intensity of infection and laying date in year 2, correlation coefficient between intensity of infection and clutch size in year 2, correlation coefficient between intensity of infection and reproductive success in year 2, and correlation coefficient between intensity of infection and body condition in year 2.

| Parasite species | r (Intensity,Laying, date) year 1 | r (Intensity, Clutch size) year 1 | r (Intensity, Reproductive success) year 1 | r (Intensity, Condition) year 1 | r (Intensity,Laying, date) year 2 | r (Intensity, Clutch size) year 2 | r (Intensity, Reproductive success) year 2 | r (Intensity, Condition) year 2 |
| --- | --- | --- | --- | --- | --- | --- | --- | --- |
| *Trichomonas gallina*e | 0.000 | 0.000 | 0.000 | . | . | . | . | . |
| Mite | 0.384 | -0.771 | -0.771 | 0.000 | 0.608 | -0.523 | -0.523 | . |
| *Trichomonas gallinae* | 0.000 | 0.000 | 0.000 | . | 0.065 | . | -0.963 | . |
| Mite | -0.561 | -0.256 | 0.209 | 0.085 | -0.055 | 0.668 | 0.660 | -0.434 |
| Ticks | -0.163 | -0.140 | 0.250 | 0.488 | -0.256 | 0.762 | 0.643 | -0.289 |
| *Dermanyssus* sp. | -0.268 | 0.291 | 0.345 | 0.081 | -0.224 | 0.400 | 0.174 | -0.188 |
| *Protocalliphora azurea* | 0.269 | -0.111 | 0.005 | 0.252 | 0.242 | -0.213 | -0.097 | -0.325 |
| *Ceratophyllus gallinae* | -0.078 | -0.395 | 0.151 | 0.140 | -0.195 | 0.160 | 0.155 | 0.391 |
| *Culicoides* sp. | -0.188 | 0.362 | 0.342 | -0.005 | -0.281 | 0.236 | 0.303 | -0.157 |
| Chewing louse | 0.327 | -0.292 | 0.122 | -0.199 | -0.071 | -0.004 | 0.015 | 0.065 |
| Feather mite | 0.031 | 0.051 | 0.089 | 0.048 | 0.123 | 0.018 | -0.018 | -0.009 |
| Louse fly | 0.000 | 0.000 | 0.000 | 0.000 | -0.400 | -0.200 | -0.400 | -0.330 |
| *Ceratophyllus gallinae* | . | . | . | . | . | . | . | . |
| *Ornithonyssus bursa* | 0.160 | -0.138 | -0.204 | 0.161 | 0.000 | 0.000 | 0.000 | 0.000 |
| *Ornithomyia avicularia* | 0.000 | 0.000 | 0.000 | 0.000 | -0.094 | -0.017 | -0.061 | 0.000 |
| *Brueelia* sp. | -0.043 | 0.072 | 0.138 | -0.259 | -0.110 | 0.066 | 0.048 | 0.053 |
| *Trouessartia crucifera* | -0.061 | 0.019 | 0.084 | -0.263 | 0.061 | -0.007 | 0.039 | 0.032 |
| *Brueelia* sp. | . | . | . | 0.297 | . | . | . | -0.041 |
| *Brueelia* sp. | . | . | . | -0.259 | . | . | . | -0.122 |
| *Trouessartia crucifera* | . | . | . | 0.148 | . | . | . | -0.232 |
| *Trouessartia crucifera* | . | . | . | -0.100 | . | . | . | 0.114 |
| Chewing louse | 0.460 | -0.279 | -0.148 | -0.182 | 0.098 | -0.250 | -0.066 | -0.234 |
| Mite | -0.030 | 0.173 | -0.128 | . | -0.391 | . | -0.552 | . |
| *Ceratophyllus gallinae* | . | . | . | . | . | . | . | . |
| *Ixodes ricinus* | 0.077 | 0.286 | 0.104 | . | 0.038 | 0.123 | 0.129 | . |
| *Protocalliphora azurea* | . | 0.287 | 0.291 | . | . | -0.124 | 0.062 | . |
| *Ceratophyllus gallinae* | 0.000 | 0.000 | 0.000 | . | . | . | . | . |
| Feather mites | 0.000 | 0.000 | 0.000 | 0.000 | 0.000 | 0.000 | 0.000 | 0.000 |
| *Carnus* sp. | -0.374 | 0.338 | 0.406 | -0.358 | 0.122 | 0.172 | 0.335 | -0.341 |
| Chewing louse | -0.134 | . | . | 0.086 | 0.007 | . | . | 0.015 |
| Ticks | -0.033 | . | . | 0.070 | -0.008 | . | . | 0.006 |
| Mite | -0.240 | 0.254 | 0.083 | -0.525 | -0.452 | 0.345 | 0.453 | . |
| *Ceratophyllus gallinae* | 0.802 | -0.022 | -0.022 | -0.500 | 0.674 | 0.858 | 0.858 | . |

Supplementary Material Table S4. Analyses of host populations based on data with an interval of sampling of 5-15 years. Replicate analyses based on data with an interval of 5-15 years between sampling events. Within subjects effects of repeated measures ANOVAs with laying date, clutch size, brood size, body condition and population density of hosts in two different study years as within subjects factors and locality identity, host identity, latitude, interval in years and temperature change (º C / year) as between subjects factors. Each effect was estimated in separates models. P-values smaller than 0.1 are shown in bold.

|  | Repeated measure | | | Locality | | | Host identity | | | Latitude | | | Interval in years | | | Temperature change  (º C/ year) | | |
| --- | --- | --- | --- | --- | --- | --- | --- | --- | --- | --- | --- | --- | --- | --- | --- | --- | --- | --- |
|  | *F* | d.f. | *P* | *F* | d.f. | *P* | *F* | d.f. | *P* | *F* | d.f. | *P* | *F* | d.f. | *P* | *F* | d.f. | *P* |
| Laying date | **7.19** | **1,19** | **0.015** | **25.56** | **15.4** | **0.003** | 1.61 | 13,6 | 0.244 | **3.65** | **1,18** | **0.072** | 1.22 | 1,18 | 0.280 | 1.65 | 1,18 | 0.214 |
| Clutch size | 0.03 | 1,20 | 0.870 | 0.73 | 15,5 | 0.713 | 1.96 | 13,7 | 0.188 | 001 | 1,19 | 0.947 | 0.13 | 1,19 | 0.720 | **25.07** | **1,19** | **<0.001** |
| Brood size | 0.01 | 1,19 | 0.945 | 1.96 | 14,5 | 0.236 | 0.21 | 12,7 | 0.990 | 2.56 | 1,18 | 0.127 | 0.39 | 1,18 | 0.542 | 0.05 | 1,18 | 0.835 |
| Body condition | 0.06 | 1,14 | 0.807 | 0.41 | 8,6 | 0.877 | 3.71 | 10.4 | 0.109 | 0.02 | 1,13 | 0.895 | 2.26 | 1,13 | 0.157 | **3.56** | **1,13** | **0.081** |
| Population density | **4.32** | **1,28** | **0.047** | **2.17** | **17,11** | **0.096** | 1.62 | 17,11 | 0.209 | 0.01 | 1,27 | 0.930 | 2.17 | 1,27 | 0.152 | 1.03 | 1,27 | 0.319 |

Supplementary Material Table S5. Analyses of determinants of parasitism based on data with an interval of sampling of 5-15 years. Within subjects effects of repeated measures ANOVAs with parasite abundance and prevalence in two different study years as within subjects factors and locality identity, parasite identity, latitude, interval in years and temperature change (º C / year) as between subjects factors. Each effect was estimated in separates models. P-values smaller than 0.1 are shown in bold.

|  | Repeated measure | | | Locality | | | Parasite identity | | | Latitude | | | Interval in years | | | Temperature change  (º C / year) | | |
| --- | --- | --- | --- | --- | --- | --- | --- | --- | --- | --- | --- | --- | --- | --- | --- | --- | --- | --- |
|  | *F* | d.f. | *P* | *F* | d.f. | *P* | *F* | d.f. | *P* | *F* | d.f. | *P* | *F* | d.f. | *P* | *F* | d.f. | *P* |
| Parasite load | **5.58** | **1,17** | **0.030** | 1.17 | 12,5 | 0.461 | 0.04 | 3,14 | 0.990 | 0.01 | 1,16 | 0.939 | 1.530 | 1,16 | 0.234 | 0.03 | 1,16 | 0.859 |
| Parasite prevalence | 2.13 | 1,29 | 0.154 | **4.67** | **15,14** | **0.003** | 0.34 | 3,26 | 0.798 | 2.42 | 1,28 | 0.131 | 0.470 | 1,28 | 0.500 | 1.400 | 1,28 | 0.250 |

Supplementary Material Table S6. Analyses of parasitism in relation to life history based on data with an interval of sampling of 5-15 years. Within subjects effects (parasite abundance/prevalence in the first and the second year) of repeated measures ANOVAs with laying date, clutch size, brood size, body condition and population density of hosts in two different study years as within subjects factors and latitude, temperature change, variation in parasitism (i.e. change in abundance and prevalence of parasites) and identity of parasite group as between subjects factors. P-values smaller than 0.1 are shown in bold.

|  | Repeated measure | | | Repeated measure * Latitude | | | Repeated measure * Temperature change | | | Repeated measure * Parasitism | | | Repeated measure * Parasite group | | |
| --- | --- | --- | --- | --- | --- | --- | --- | --- | --- | --- | --- | --- | --- | --- | --- |
|  | *F* | d.f. | *P* | *F* | d.f. | *P* | *F* | d.f. | *P* | *F* | d.f. | *P* | *F* | d.f. | *P* |
| Parasite abundance | | |  |  |  |  |  |  |  |  |  |  |  |  |  |
| Laying date | **6.24** | **1,10** | **0.031** | **3.88** | **1,10** | **0.077** | 0.398 | 1,10 | 0.542 | 3.16 | 1,10 | 0.106 | **9.64** | **3,10** | **0.003** |
| Clutch size | **12.20** | **1,10** | **0.006** | **12.49** | **1,00** | **0.005** | **9.85** | **1,10** | **0.011** | **6.80** | **1,10** | **0.026** | **5.71** | **3,10** | **0.015** |
| Brood size | **18.65** | **1, 9** | **0.002** | **23.72** | **1,9** | **0.001** | **13.53** | **1,9** | **0.005** | **7.83** | **1,9** | **0.021** | **3.15** | **3,9** | **0.079** |
| Body condition | **48.16** | **1,4** | **0.002** | **50.69** | **1,4** | **0.002** | **55.86** | **1,4** | **0.002** | **9.25** | **1,13** | **0.038** | **34.05** | **3,4** | **0.003** |
| Population density | **4.80** | **1,10** | **0.053** | **4.92** | **1,10** | **0.051** | 0.84 | 1,10 | 0.380 | 0.45 | 1,10 | 0.517 | **4.31** | **3,10** | **0.034** |
| Parasite prevalence | | |  |  |  |  |  |  |  |  |  |  |  |  |  |
| Laying date | **9.93** | **1,14** | **0.007** | **6.35** | **1,14** | **0.025** | 0.11 | 1,14 | 0.111 | 1.20 | 1,14 | 0.291 | **5.33** | **3,14** | **0.012** |
| Clutch size | **4.37** | **1,15** | **0.054** | **4.00** | **1,15** | **0.064** | **5.33** | **1,15** | **0.036** | 0.05 | 1,15 | 0.818 | 1.33 | 3,15 | 0.301 |
| Brood size | 2.24 | 1,14 | 0.156 | 2.23 | 1,14 | 0.157 | 1.96 | 1,14 | 0.184 | 1.98 | 1,14 | 0.181 | 0.93 | 3,14 | 0.453 |
| Body condition | 2.41 | 1,11 | 0.149 | 3.07 | 1,11 | 0.107 | **5.29** | **1,11** | **0.042** | 0.97 | 1,11 | 0.347 | 2.37 | 3,11 | 0.126 |
| Population density | 1.03 | 1,23 | 0.321 | 1.31 | 1,23 | 0.264 | 0.26 | 1,23 | 0.613 | 0.38 | 1,23 | 0.542 | **2.71** | **3,23** | **0.069** |
